# Empagliflozin does not prevent progression of Dent's disease type 1 in a mouse model

**DOI:** 10.1113/EP092736

**Published:** 2025-07-22

**Authors:** Elise de Combiens, Nadia Frachon, Yohan Bignon, Marc Fila, Clément Brossard, Perrine Frère, Stéphane Lourdel

**Affiliations:** ^1^ Centre de Recherche des Cordeliers, INSERM, Sorbonne Université, Université Paris Cité Paris France; ^2^ CNRS EMR 8228 Paris France; ^3^ Department of Biomedical Sciences University of Lausanne Lausanne Switzerland; ^4^ Néphrologie pédiatrique CHU Arnaud de Villeneuve Montpellier France; ^5^ Sorbonne Université, INSERM, Unité mixte de Recherche 1155, Kidney Research Centre, AP‐HP, Hôpital Tenon Paris France

**Keywords:** Dent's disease, empagliflozin, proximal tubule, SGLT2 inhibitor

## Abstract

Dent's disease is a rare inherited renal disorder characterized by generalized proximal tubule dysfunction with low molecular weight proteinuria, hypercalciuria, and urinary loss of other solutes. The disease is progressive and leads to chronic kidney disease. To study the mechanisms involved in its progression, we generated a knock‐in mouse model displaying a classical Dent's disease type 1 phenotype. Currently, no targeted therapy exists for Dent's disease; treatment strategies primarily aim to slow the progression of specific clinical aspects. Accordingly, empagliflozin [a sodium–glucose cotransporter 2 (SGLT2) inhibitor] known to exert nephroprotective effects and to slow down the decrease of the glomerular filtration rate in diabetic and non‐diabetic patients with chronic kidney disease, was administered to the knock‐in mice. We demonstrated that empagliflozin administration reduces renal and urinary levels of the marker of tubular damage, Lipocalin‐2 (LCN2). However, we observed that this preventive treatment does not alleviate low molecular weight proteinuria, hypercalciuria, inflammation, renal fibrosis or the decline of the glomerular filtration rate. Overall, our findings suggest that SGLT2 inhibition with empagliflozin does not prevent the progression of Dent's disease type 1 towards chronic kidney disease.

## Abstract (highlights)

- **What is the central question of the study?**
  Can empagliflozin prevent the progression of Dent's disease type 1, a rare inherited renal disorder that frequently leads to chronic kidney disease?
- **What is the main finding and its importance?**
  Administration of empagliflozin to a model of Dent's disease type 1 reduced the expression of LCN2, which might be involved in the development of fibrosis. Surprisingly, fibrosis was not reduced, suggesting that it might be involved in other mechanisms of the disease progression. Furthermore, administration of empagliflozin for 8 months did not seem to reduce the major clinical features of Dent's disease type 1.

## 1 INTRODUCTION

Dent's disease is a rare inherited renal disorder characterized by low molecular weight proteinuria, often accompanied by hypercalciuria, nephrocalcinosis or nephrolithiasis and the urinary loss of solutes, such as amino acids, phosphate, magnesium or glucose, all linked to generalized proximal tubule dysfunction. The disease is progressive and can often lead to end‐stage renal failure, necessitating dialysis or kidney transplantation (Blanchard et al., 2016; Devuyst & Thakker, 2010; Mansour‐Hendili et al., 2015).

The disease mainly affects men, because the genes involved are located on the X chromosome. The disease is genetically heterogeneous; ∼65% of patients affected by Dent's disease type 1 harbour inactivating mutations of *CLCN5*, encoding the 2Cl^−^–H^+^ exchanger ClC‐5. ClC‐5 is predominantly expressed in early endosomes of proximal tubule cells, where it plays a crucial role in the endocytosis of low molecular weight proteins by optimizing the endosomal ionic content, a necessary step for the proper trafficking of endocytotic vesicles and the recycling of transporters and receptors at the plasma membrane (Günther et al., 1998; Piwon et al., 2000).

Studies of cellular and molecular mechanisms involved in Dent's disease type 1 recently included the use of a ClC‐5 knock‐in (KI) mouse model harbouring a pathogenic missense mutation (*Arg340lys* or *N340K*) of *Clcn5* (Tosetto et al., 2006) representative of ClC‐5 mutants retained in the endoplasmic reticulum. In this model, data have demonstrated that knock‐in mice display a classical Dent's disease phenotype accompanied by an alteration of the endo‐lysosomal pathway in proximal tubule cells. Additionally, age‐related progression of their phenotype leads to chronic renal alterations, as demonstrated by a decrease of their estimated glomerular filtration rate (eGFR) and an increase of renal fibrosis and apoptosis (Sakhi et al., 2024).

Current treatments aim to reduce the progression of some clinicals aspects, such as nephrocalcinosis (Devuyst & Thakker, 2010; Gianesello et al., 2017; Mansour‐Hendili et al., 2015). Recently, sodium–glucose cotransporter 2 (SGLT2) inhibitors, such as empagliflozin, canagliflozin and dapagliflozin, which were developed initially to treat patients with type 2 diabetes, have been shown to slow down the progression of chronic kidney disease (CKD). These molecules enhance urinary glucose excretion and thus reduce hyperglycaemia by reducing SGLT2‐mediated proximal tubule reabsorption that occurs at the apical membrane of proximal epithelial cells and results in the reabsorption of ∼90% of the filtered glucose (Vallon et al., 2011).

Recent data have demonstrated that these inhibitors display significant benefits on renal outcomes in diabetic and non‐diabetic patients, with or without albuminuria, suffering from CKD, including a slower rate of decline in eGFR or in the progression of albuminuria (Bailey et al., 2022; Delanaye & Scheen, 2023; Nakhleh et al., 2024; Podestà et al., 2023). Additionally, studies performed with diabetic patients with CKD and non‐diabetic rats with CKD have reported that empagliflozin or canagliflozin treatment produces a reduction of the circulating levels of markers related to inflammation, fibrosis and oxidative stress (Chen et al., 2023; Heerspink et al., 2019; Li et al., 2019).

Furthermore, empagliflozin, canagliflozin or dapagliflozin reduced the prevalence of nephrolithiasis in men with diabetes, and phlorizin reduced the formation of calcium oxalate kidney stones and the expression of the tubular injury markers KIM‐1 and LCN2 (known to be involved in fibrosis) in the cortex of rats with nephrolithiasis (Anan et al., 2022).

Considering their multiple positive effects, SGLT2 inhibitors might represent a potential therapeutic interest for Dent's disease patients. Accordingly, the aim of this study was to investigate the effects of 8 months of administration of empagliflozin in ClC‐5 *N340K* mice on their renal phenotype.

Our results demonstrate that, interestingly, empagliflozin reduces the renal expression and urinary excretion of the LCN2 but does not reduce renal fibrosis, suggesting another role of LCN2 in the progression of Dent's disease. However, empagliflozin does not significantly improve major clinical features of Dent's disease, nor GFR decline.

## 2 MATERIALS AND METHODS

### 2.1 Ethical approval

The scientific project and the experimental procedures were favourably evaluated by the Charles Darwin Ethics Committee for animal experimentation [permit 03‐SL/(2)#44938].

### 2.2 Animals

The transgenic mouse strain ClC‐5 *N340K* was designed and created as mentioned by Sakhi et al. (2024). Mice were housed in the Centre d'Exploration Fonctionnelle (UMRS1138, Paris, France; permit B75‐06‐12). Animals were housed in a climate‐controlled facility with a 12 h–12 h light–dark cycle and had free access to food, deionized water and housing. For the experiments, male mice only were divided into four groups: untreated wild‐type (WT), untreated knock‐in (KI), empagliflozin‐treated WT and empagliflozin‐treated KI. Empagliflozin was incorporated in the standard food (SAFE‐A04 powder chow), 180 mg/kg of chow, from 2 to 10 months of age; this dose is equivalent to 24 mg/kg mouse body weight based on a daily consumption of 4 g of chow each day.

### 2.3 Metabolic studies

Metabolic studies were performed on 10‐month‐old male mice, housed in individual metabolic cages (Tecniplast, Decines Charpieu, France), after an adaptation period of 3 days. Urine was collected during a period of 24 h under water‐saturated mineral oil. Venous blood was collected from the retro‐orbital plexus of conscious mice at the end of the experimental period. Urinary Ca^2+^, Mg^2+^, phosphate, creatinine and plasma urea and creatinine contents were determined using a Konelab 20I analyser (ThemoFisher Scientific).

### 2.4 Real‐time quantitative PCR analyses, RNA extractions and reverse transcription

Kidneys were harvested from anaesthetized mice by intraperitoneal injection of ketamine and xylazine (Coveto), then frozen in liquid nitrogen. Reverse transcription (RT) was performed using the first strand cDNA synthesis kit for RT‐PCR (Roche Diagnostics). For the real‐time quantitative PCR experiments, the amount of PCR product in each sample was calculated as a percentage based on an RNA standard curve, which was established using serial dilutions of a mouse whole‐kidney cDNA stock solution. These values were then normalized to the amount of PCR product for mRNA of the housekeeping gene ribosomal protein L26 (*Rpl26*). Primers list is available in Table 1.

**TABLE 1:** List of primers used for quantitative PCR

Gene	Forward	Reverse
*Acta2*	CGTTACTACTGCTGAGCGTGA	AACGTTCATTTCCGATGGTG
*Col1a1*	CCCCGGGACTCCTGGACTT	GCTCCGACACGCCCTCTCTC
*Col3a1*	CTGGACCCCAGGGTCTTC	CATCTGATCCAGGGTTTCCA
*Col4a1*	AACAACGTCTGCAACTTCGC	TGACTGTGTACCGCCATCAC
*Fn1*	CCTACGGCCACTGTGTCACC	AGTCTGGGTCACGGCTGTCT
*Slc5a2*	ATTGTCTCGGGCTGGTATTG	CAGGTAGCCACACAAGATGC
*Rpl26*	GCTAATGGCACAACCGTC	TCTCGATCGTTTCTTCCTTGTAT

### 2.5 Western blotting and Coomassie staining

Experiments were performed as previously detailed (Sakhi et al., 2024). Briefly, mouse kidneys were snap‐frozen into liquid nitrogen, mechanically homogenized and incubated in an ice‐cold lysis buffer supplemented with antiproteases. Proteins were dosed using a BCA Protein Assay Kit (ThermoFischer Scientific). About 20 µg of proteins were denaturated in a 2× Laemmli buffer for 10 min at 95°C. Proteins were then separated on 4%–12% or 4%–20% bis‐acrylamide gels, transferred onto nitrocellulose membranes, blocked and incubated with primary and horseradish peroxidase‐conjugated secondary antibodies using a Tris‐Buffered Saline supplemented with 5% non‐fat milk proteins and 0.05% Tween20. Antibodies [rabbit anti‐SGLT2, Proteintech (24654‐1‐AP); goat anti‐LCN2, R&D Systems (AF1857); and mouse anti‐KIM‐1 ABCAM (ab233720)] were used for detection of the proteins.

### 2.6 Histochemistry and immunofluorescence

Mice were anaesthetized by intraperitoneal injection of ketamine and xylazine (Coveto), and kidneys were fixed by transcardial perfusion with 4% paraformaldehyde‐containing PBS, harvested, washed in ice‐cold PBS and paraffin embedded. At the time of the experiment, 7‐ or 5‐µm‐thick sagittal sections were cut using an RM2145 microtome (Leica Biosystems, Nanterre, France). For analysis of calcium deposits, Alizarin Red staining was performed following the provider's recommendations (Sigma Aldrich). Sirius Red staining was performed following the provider's recommendations (cliniscience).

A citrate pH 6 solution used for antigen retrieval. Tissues were blocked with 10% serum and 1% bovine serum albumin. Kidney sections were then incubated with primary antibody [rabbit anti‐CD3, Abcam (ab16669); or rabbit anti‐SGLT2, Proteintech (24654‐1‐ap)], then with AlexaFluor or Phalloidin‐FITC and 4′,6‐diamidino‐2‐phenylindole (DAPI).

Images were acquired using an AxioScanZ1 slide scanner (Leica), Vectra Polaris (Akoya) or LSM710 confocal laser scanning microscope (Zeiss, Oberkochen, Germany).

## 3 RESULTS

### 3.1 Effect of empagliflozin on the phenotype of the KI mice

**FIGURE 1:**
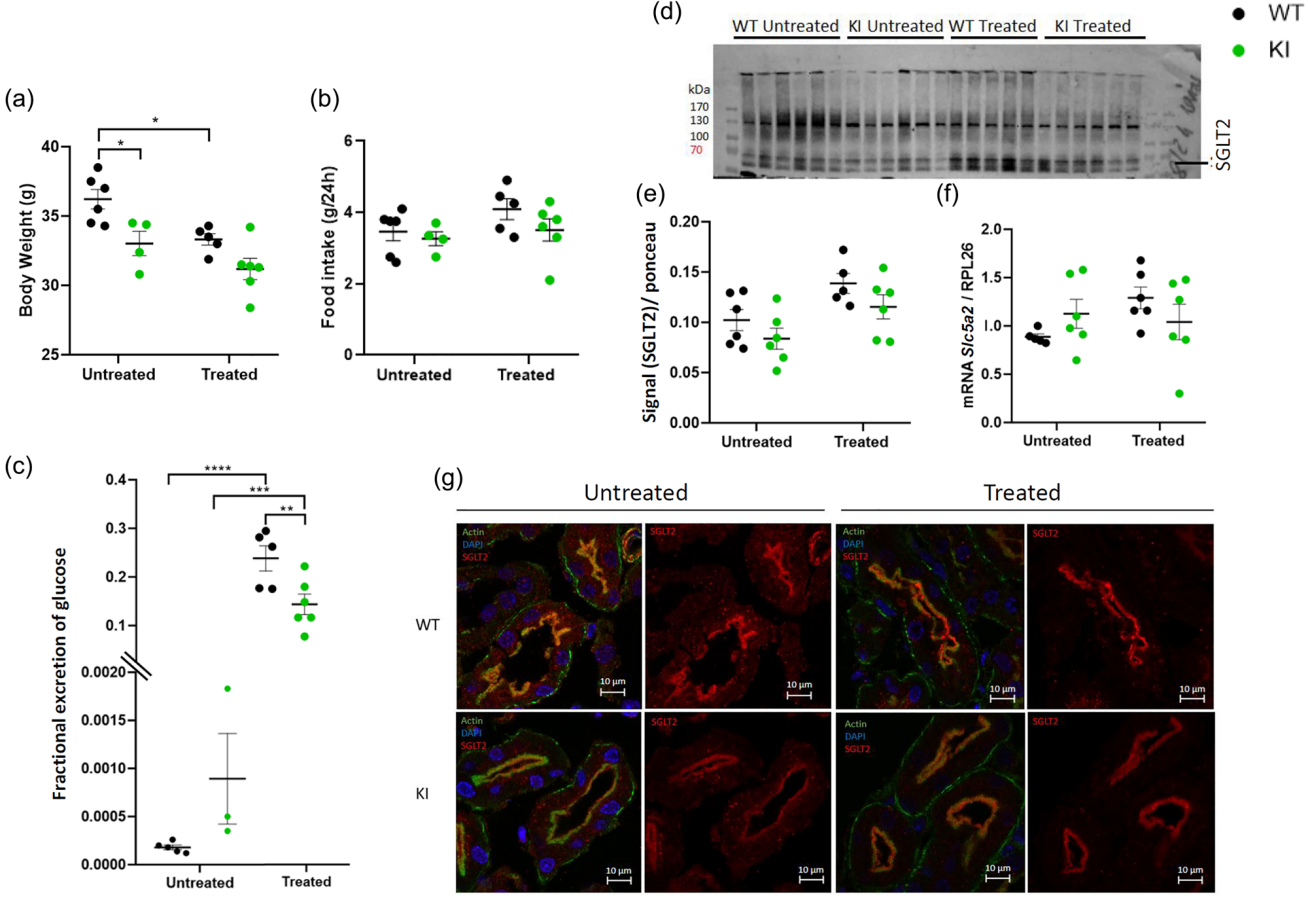
Effects of empagliflozin on body weight, food intake, glucose excretion and SGLT2 expression on WT and KI mice. (a,b) Body weight and food intake at 10 months. (c) 24 h excretion of glucose (*n *= 3/6). (d,e) Western blot of SGLT2 and its quantification (*n* = 5/6). (f) *Slc5a2* mRNA expression in kidney normalized by *Rpl26* (*n *= 5/6). (g) Immunofluorescence of SGLT2 in kidney, using DAPI to stain the nucleus and actin to highlight the brush border of proximal tubule cells (*n* = 2). Analyses were made by two‐way ANOVA followed by multiple comparisons and a Bonferroni correction. Results are represented as individual values with means ± SEM. ^*^ *P *< 0.05, ^**^ *P *< 0.01, ^***^ *P* < 0.001 is the difference between KI and WT mice. Abbreviations: KI, knock‐in; SGLT2, sodium–glucose cotransporter 2; WT, wild‐type.

Empagliflozin was administered in the standard diet of 2‐month‐old WT and KI male mice for 8 months. Samples from untreated groups were from mice already used in past studies (Sakhi et al., 2024) owing to a very low birth rate of these mice. Body weight measurements at 10 months demonstrated that untreated KI mice had significantly lower body weight compared with WT mice (Figure 1a). In contrast to KI mice, empagliflozin treatment resulted in a significant lowering of body weight of the WT mice after 8 months. These initial differences and variations of body weight were not associated with variation in food intake (Figure 1b), indicating that there was no variation in empagliflozin intake by the mice throughout the experiment.

Empagliflozin is a specific inhibitor of the Na^+^–glucose cotransporter SGLT2, which is expressed at the apical membrane of proximal tubule cells. Our data demonstrated that SGLT2 was still expressed in proximal tubule cells of KI animals, although its protein expression and apical localization were decreased in comparison to those of WT mice (Figure 1g). Empagliflozin did not significantly change *Slc5a2* gene expression or SGLT2 protein expression and localization (Figure 1d–g). Untreated KI animals presented a slightly but not statistically significant increase in glucose excretion (owing to small sample size). However, as expected, empagliflozin administration resulted in a significant increase of glucose excretion in both WT and KI animals (Figure 1c).

**FIGURE 2:**
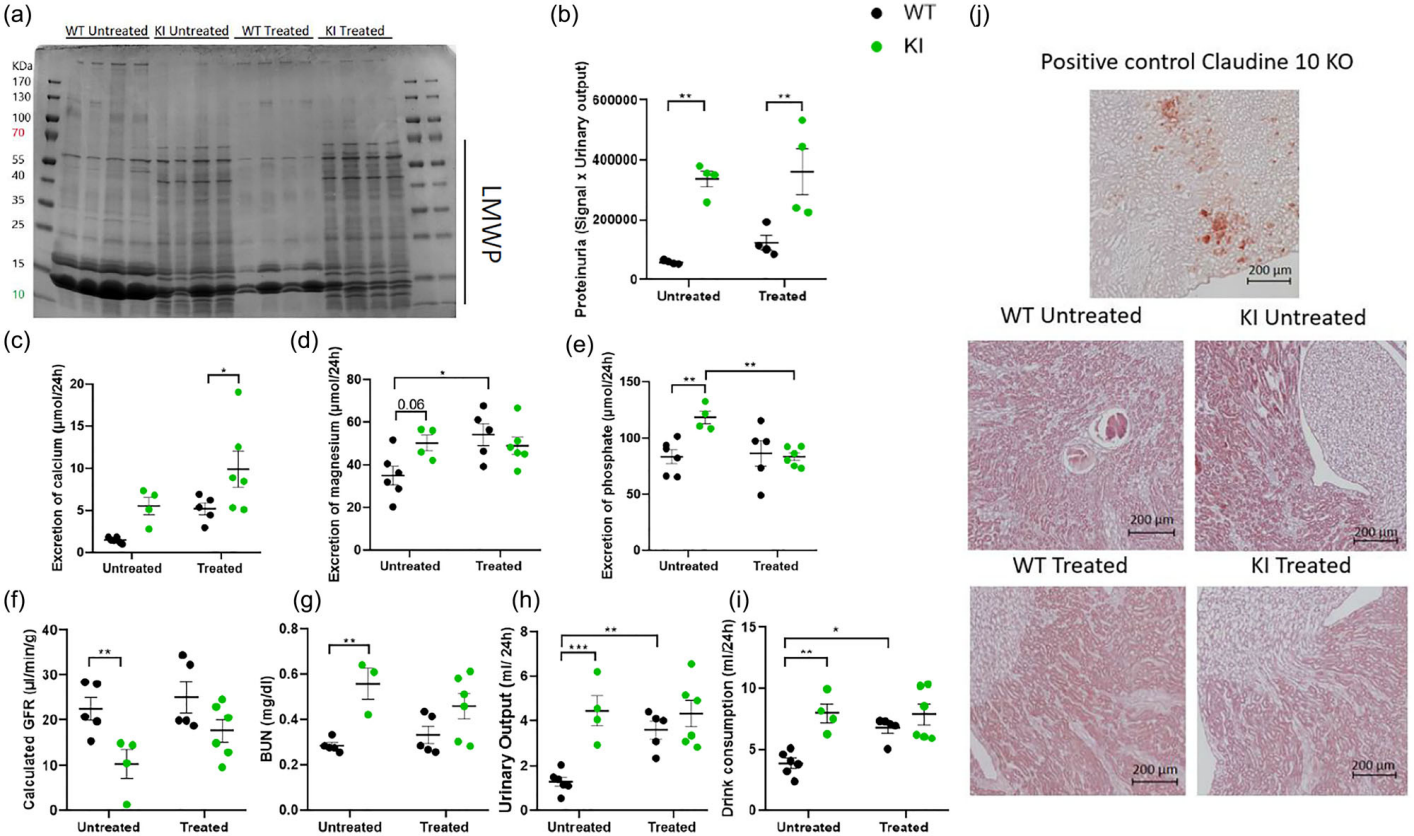
Effect of empagliflozin on the renal function of the mice. (a) Coomassie gel staining of urinary proteins from 10‐month‐old animals. (b) Quantification of the Coomassie gel staining normalized by urinary output (*n *= 4). (c–e) 24 h excretion of calcium (c), magnesium (d) and phosphate (e). (f) Calculated GFR from 10‐month‐old mice (*n = *4–6). (g) BUN calculated by the formula: plasmatic urea (in milligrams per decilitre) × 2.8 (*n = *3–6). (h,i) 24 h urinary output and drink consumption in 10‐month‐old animals (*n *= 4–6). (j) Alizarin Red‐stained kidney sections from 10‐month‐old animals. Claudin 10 KO mouse is a positive control of nephrocalcinosis shown by red deposits (*n *= 2). Zooms are representative of the whole slice. Analyses were made by two‐way ANOVA followed by multiple comparisons and a Bonferroni correction. Data are represented by individual values with the mean ± SEM. ^*^ *P *< 0.05, ^**^ *P *< 0.01, ^***^ *P* < 0.001 is the difference between KI and WT mice. Abbreviations: BUN, blood urea nitrogen; GFR, glomerular filtration rate; KI, knock‐in; KO, knock‐out; WT, wild‐type.

Untreated KI mice exhibited low molecular weight proteinuria, which was not decreased by empagliflozin administration (Figure 2a,b). Untreated KI mice showed 5‐fold higher calcium excretion compared with untreated WT mice. Additionally, empagliflozin administration increased calcium excretion in WT mice and even more in KI animals (Figure 2c). A similar trend of increased magnesium excretion was also noticed in untreated KI animals compared with untreated WT, but empagliflozin administration significantly elevated magnesium excretion in WT animals only (Figure 2d). Furthermore, untreated KI animals showed a significantly increased phosphate excretion compared with untreated WT, which was normalized by empagliflozin administration in KI (Figure 2e).

We also calculated the glomerular filtration rate (GFR) of the WT and KI animals using urinary and plasma creatinine concentrations. As published in our previous study with this model (Sakhi et al., 2024), the eGFR of untreated KI mice was significantly decreased in comparison to untreated WT mice. Despite an intriguing tendency towards higher eGFR in empagliflozin‐treated KI mice compared with untreated KI mice, it is interesting to note that the eGFR of KI mice was not different from those of WT mice during empagliflozin treatment (Figure 2f). Blood urea nitrogen, which was higher in untreated KI mice, also showed no difference between WT and KI with empagliflozin administration, suggesting a sight drop in plasma urea concentration in KI mice receiving empagliflozin, although no significant difference could be shown compared with untreated KI mice (Figure 2g).

Untreated 10‐month‐old KI mice were polyuric (Figure 2h), accompanied by increased water consumption in comparison to untreated WT mice (Figure 2i). Owing to the diuretic effect of SGLT2 inhibitors, treated WT mice also presented a significantly increased urinary output and water consumption. In contrast, empagliflozin did not increase polyuria further in KI mice, because it was already elevated, resulting in no variation in their water consumption (Figure 2i).

Given that hypercalciuria was observed in KI animals, we checked for possible presence of nephrocalcinosis, but no signs of this disorder were observed (zooms are representative of the whole‐kidney slices) in either untreated or treated KI mice (Figure 2j).

Overall, our results demonstrate that empagliflozin treatment did not significantly improve the clinical symptoms in KI mice and had no effect on the eGFR.

### 3.2 Empagliflozin does not decrease renal fibrosis and inflammation

**FIGURE 3:**
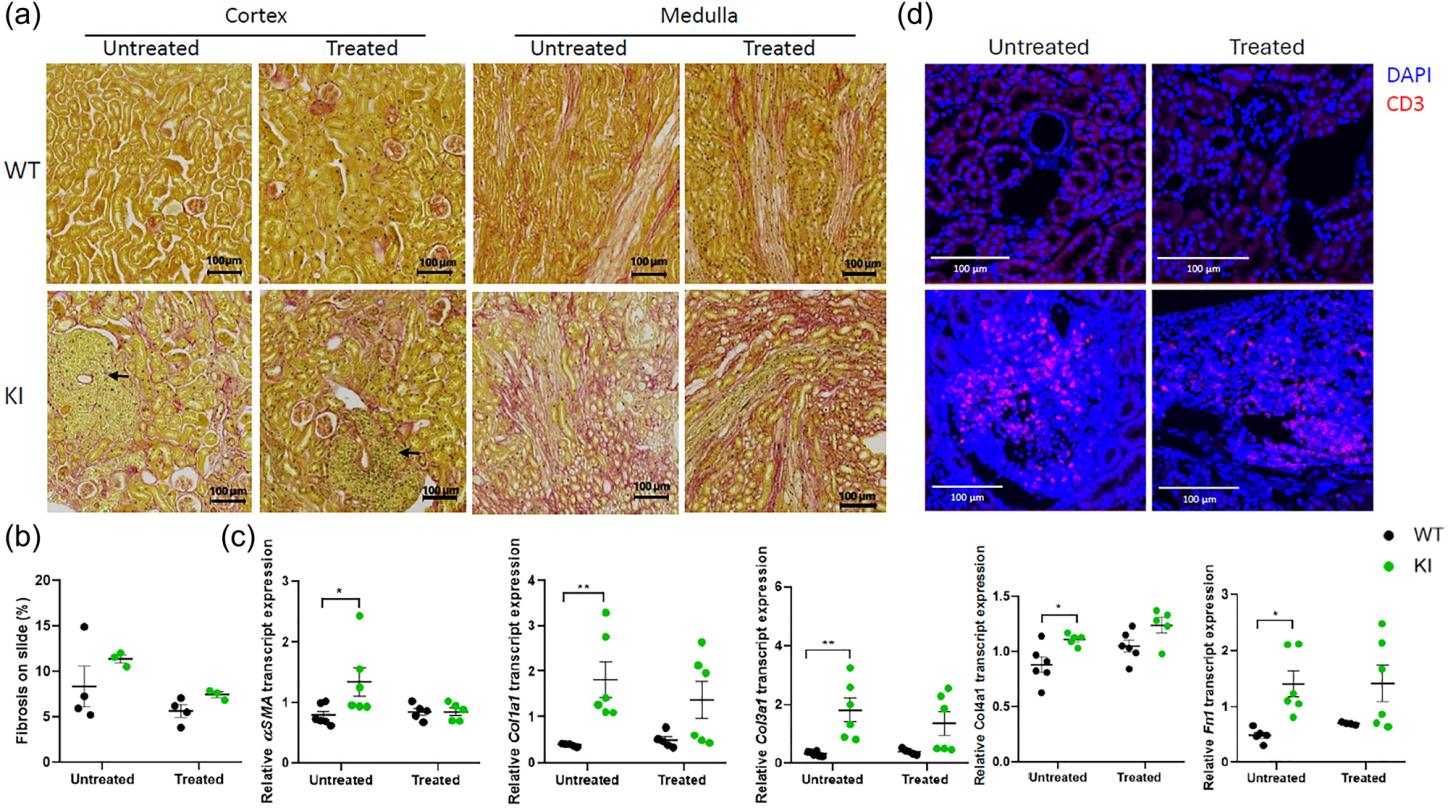
Effects of empagliflozin on renal fibrosis and inflammation in the KI mouse model of Dent's disease type 1. (a) Sirius Red staining on full kidney section. Renal fibrosis appears in red; arrow shows immune cell infiltrate. (b) Quantification of fibrosis on kidney slices from 10‐month‐old animals measured by pink area in cortex/total area (mean of three slices per animal; *n = *4). (c) RT‐qPCR analysis of *αSma*, *Col1a1*, *Col3a1*, *Col4a1* and *Fn1* gene markers for renal fibrosis in 10‐month‐old animals (*n *= 6). (d) Immunofluorescence of CD3 (*n* = 3–7). Analyses were made by two‐way ANOVA followed by multiple comparisons and a Bonferroni correction. Results are represented as individual values with means ± SEM. ^*^ *P *< 0.05, ^**^ *P *< 0.01, ^***^ *P* < 0.001 is the difference between KI and WT mice. Abbreviations: CD3, Cluster of differentiation 3, KI, knock‐in; WT, wild‐type.

Chronic kidney diseases, such as Dent's disease type 1, are frequently associated with renal fibrosis. Kidney cross‐sections of 10‐month‐old untreated KI mice revealed prominent fibrosis, which was not alleviated in KI mice exposed to empagliflozin (Figure 3a,b). We also quantified the transcript levels of several markers for renal fibrosis. Consistent with increased fibrosis observed in kidneys sections from untreated KI mice, we observed significantly higher expression of *ɑSma*, *Col1a1*, *Col3a1*, *Col4a1* and *Fn1* in kidneys of untreated KI mice and empagliflozin‐treated KI mice (Figure 3c). Sirius Red staining also revealed immune infiltrates around vessels of KI mice (shown by arrows), which were still present with empagliflozin. Immunofluorescence of CD3 also confirmed inflammation around vessels of KI mice with or without empagliflozin (Figure 3d). Empagliflozin does not appear to induce changes in renal histological structures (neither protection nor worsening of tubular dilatation). Overall, these data indicate that empagliflozin treatment does not reduce renal fibrosis or inflammation in KI animals.

### 3.3 Empagliflozin induces a reduction of the urinary excretion and of the renal expression of lipocalin‐2

**FIGURE 4:**
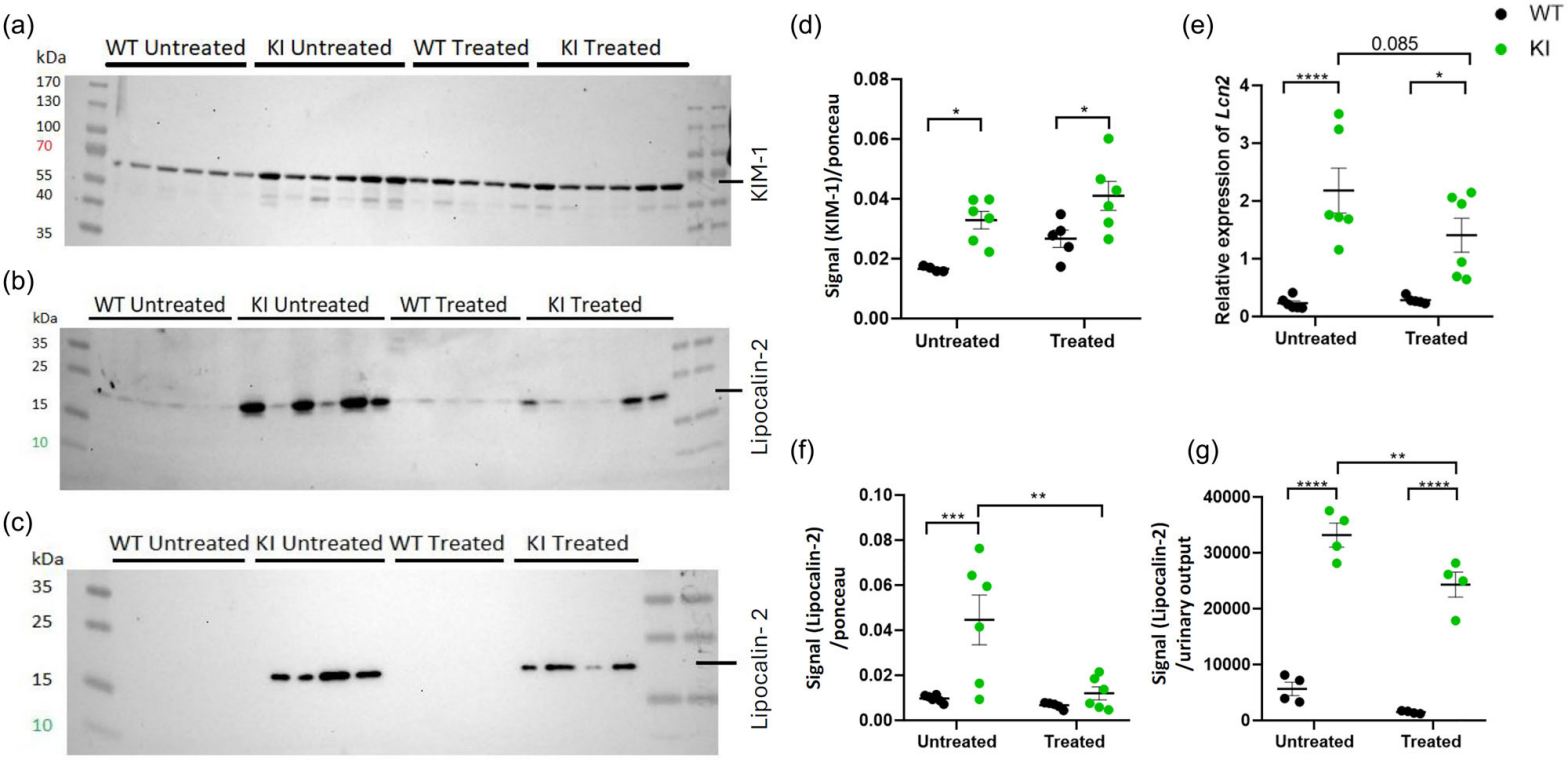
Impact of empagliflozin on the renal abundance and on the urinary excretion of Lipocalin‐2 (LCN2) and KIM‐1 in the KI mice. (a) Western blot analysis of KIM‐1 expression in the kidneys of 10‐month‐old mice. (b) Western blot analysis of LLCN2 expression in the kidneys of 10‐month‐old mice. (c) Western blot analysis of LCN2 urinary excretion in 10‐month‐old mice. (d) Quantification of KIM‐1 in the kidneys (*n *= 5–6). Signals have been normalized by Ponceau staining. (e) RT‐qPCR analysis of *Lcn2* in 10‐month‐old animals (*n *= 6). (f,g) Quantification of LCN2 in kidney (*n *= 5–6; f) and in urine (*n *= 4; g). Signals have been normalized by Ponceau staining and urinary output, respectively. Analyses were made by two‐way ANOVA followed by multiple comparisons and a Bonferroni correction. Results are represented as individual values with means ± SEM. ^*^ *P *< 0.05, ^**^ *P *< 0.01, ^***^ *P* < 0.001 is the difference between KI and WT mice. Abbreviations: KI, knock‐in; KIM‐1, kidney Injury Molecule 1, WT, wild‐type.

We observed that KIM‐1, a well‐known marker of early tubular damage, was increased in kidneys of KI mice, and empagliflozin did not reduce its expression in comparison to the respective WT condition (Figure 4a,d). Additionally, increased expression of LCN2, another tubular injury marker usually associated with the progression CKD (Buonafine et al., 2018), was also noticed in their kidneys (Figure 3b–f). As published in our previous study (Sakhi et al., 2024), untreated 10‐month‐old animals demonstrated increased urinary excretion of LCN2 (Figure 4c–g). However, administration of empagliflozin induced a significant decrease of the urinary loss and renal protein expression of LCN2 in the KI animals.

Together, these results demonstrate that empagliflozin attenuates the urinary and kidney levels of LCN2 observed in our model of Dent's disease but has no effect on KIM‐1.

## 4 DISCUSSION

Progression to CKD is the main outcome risk for patients with Dent's disease type 1. Currently, therapies focus primarily on limiting clinical symptoms attributable to the urinary loss of certain filtered solutes. Interestingly, inhibition of the Na^+^–glucose cotransporter SGLT2, localized at the apical surface of proximal tubule cells, by drugs such as empagliflozin has been shown by several clinical trials to exert beneficial nephroprotective benefits in diabetic and non‐diabetic patients with CKD by alleviating several renal outcomes, such as the decrease in their GFR, albuminuria, or decreased need for dialysis or kidney transplantation (Delanaye & Scheen, 2023).

Furthermore, recent studies have demonstrated that empagliflozin decreases renal fibrosis and nephrolithiasis (Chen et al., 2023; Heerspink et al., 2019). Both phenomena are observed in a mouse model and some patients with Dent's disease (Blanchard et al., 2016; Sakhi et al., 2024). In the light of these positive effects, we performed experiments to evaluate the effects of empagliflozin on renal histology and function in KI mice, which exhibit typical clinical features of Dent's disease type 1, including the progression to CKD (Sakhi et al., 2024). Owing to a very small generation of KI mice (possibly owing to perinatal mortality as mentioned by Yadav et al. (2022) in their knock‐out *Clcn5* mouse model (Yadav et al., 2022), the untreated mouse samples that we obtained previously (Sakhi et al., 2024) were used to perform the present experiments.

Interestingly, we demonstrated in KI animals a significant effect of empagliflozin treatment on LCN2, a marker of tubular damage. We observed decreased kidney protein expression and urinary excretion of LCN2 in the transgenic mice, consistent with findings from other studies (Ahmed et al., 2021; Anan et al., 2022). LCN2 is not only a tubular lesion marker but also a pro‐fibrosis and pro‐inflammatory key player involved in the development of renal damage, in particular fibroblast activation (Bienaimé et al., 2016; Buonafine et al., 2018; Tarjus et al., 2015; Viau et al., 2010). However, this effect is not visible in the expression of KIM‐1, indicating a specific effect of empagliflozin on LCN2. Surprisingly, despite a significant reduction in its expression, no improvement in renal fibrosis, inflammation or tubular appearance was observed, suggesting that LCN2 plays another role in the pathological mechanisms involved in progression of Dent's disease.

Furthermore, the amount of LCN2 in urine is correlated with the rate of development of CKD. This reduction might suggest that the decline in renal function is slowed in treated KI animals, with the average GFR slightly above the untreated group. However, no significant change could be demonstrated, probably owing to the low availability of transgenic animals.

In addition, empagliflozin treatment reduced weight gain in 10‐month‐old WT mice and slightly for KI mice, consistent with literature reports in diabetic patients that attribute this weight loss to treatment‐induced glycosuria (Scheen & Delanaye, 2021). However, KI mice had lower initial body weight owing to lower fat mass (Sakhi et al., 2024).

Furthermore, KI mice initially exhibited low glycosuria, which might indicate that weight loss relative to urinary glucose wasting was already occurring in this group without empagliflozin.

In our KI model, empagliflozin treatment did not improve low molecular weight proteinuria (as observed in CKD non‐diabetic patients treated with dapagliflozin; Cherney et al., 2020). The KI mice presented basic hypercalciuria, with a urinary calcium level five times higher than the mouse control, which is consistent with observations made in other models of Dent's disease (Piwon et al., 2000; Sakhi et al., 2024; Wang et al., 2000). Surprisingly, empagliflozin treatment increased urinary calcium excretion in both groups by ∼5 µmol/day. As discussed by Edwards and Bonny (2018), a likely cause of increased calcium excretion is an elevated tubular flow resulting from osmotic diuresis (which might arise from inhibition of SGLT2), which would reduce paracellular calcium reabsorption in the proximal tubule by decreasing the calcium gradient between the tubule and medullary interstitium.

Hypercalciuria is a risk factor of nephrocalcinosis; however, no signs of nephrocalcinosis were observed, even at 10 months. Some literature reports a lower risk of nephrolithiasis in patients treated with an SGLT2 inhibitor by potentially inducing osmotic diuresis, which could explain the absence of this disorder in WT and KI mice during empagliflozin treatment despite their hypercalciuria. Additionally, these mice had a more acidic urinary pH (Sakhi et al., 2024), which might contribute to a nephroprotective effect by preventing kidney stone formation (Anan et al., 2022; Kanbay et al., 2024; Sayer et al., 2004).

Tubular phosphate reabsorption is mostly ensured by the proximal tubule, which can explain the hyperphosphaturia observed in Fanconi syndrome. Hence, in KI mice and in one previous mouse model knock‐out for *Clcn5*, the amount of Sodium‐dependent phosphate transport protein 2A (NaPi‐2a) at the apical membrane is reduced owing to impaired recycling of endosomes, leading to phosphaturia (Piwon et al., 2000; Sakhi et al., 2024). The same mechanisms might occur for glucose excretion. However, the expression of glucose and phosphate transporters at the apical membrane of proximal cells is not completely abolished in KI mice (Sakhi et al., 2024). We hypothesize that SGLT2 inhibition in KI mice somewhat increases the availability of the sodium gradient used by NaPi2a, thus increasing phosphate reabsorption in the treated KI group, therefore explaining their normalized phosphate excretion (Vinke et al., 2019). However, in WT mice, the expression of NaPi2a is not altered, and phosphate is reabsorbed even in the absence of empagliflozin, without any impact on it during SGLT2 inhibitor administration.

In contrast, empagliflozin increased the urinary wasting of magnesium in WT mice, suggesting that SGLT2 inhibition interferes with magnesium reabsorption in tubules. If the same alteration in the renal handling of magnesium occurs spontaneously in KI mice, it would explain their spontaneous urinary wasting of magnesium and the absence of an additive effect during empagliflozin treatment. However, given that the regulation of magnesium reabsorption is ensured by different tubular segments, such as the thick ascending limb and the distal convoluted tubule, the involvement of a possible compensatory mechanism by these segments cannot be ruled out.

In WT treated animals, we observed an increase in urinary output, probably explained by the significant glucose loss from SGLT2 inhibition, which might promote osmotic diuresis. In untreated KI animals, as observed in patients, diuresis was already increased, which is comparable to what is observed in some patients with Dent's disease. Alternatively, given that SGTL2 expression is already reduced in untreated KI mice, which initially exhibit glycosuria, the effects of empagliflozin could be less pronounced in KI mice than in WT mice.

Furthermore, treatment did not induce a significative change in average GFR in either WT or KI animals, nor in the amount of renal fibrosis and inflammation at 10 months, despite a seemingly small difference on average for the GFR and blood urea nitrogen and a reduction in lipocalin‐2 production. In accordance with these observed data, other published data have also shown an absence of effect induced by SGLT2 inhibitors on different parameters, such as proteinuria, GFR and the level of renal fibrosis. For example, Zhang et al. (2016) demonstrated that in mice with chronic renal failure induced by five‐sixths nephrectomy, canagliflozin treatment worsened proteinuria and showed no improvement in calculated GFR or renal fibrosis (Cherney et al., 2020; Komala et al., 2014; Zhang et al., 2016).

Some studies have highlighted that the dose of SGLT2 inhibitor is an important parameter in treatment outcomes (Lin et al., 2023). In addition, some research suggests that the effects of SGLT2 inhibitors could be increased 10‐fold in the presence of renin–angiotensin–aldosterone system inhibitors (Rastogi et al., 2024; Scheen & Delanaye, 2022). Given that renin–angiotensin–aldosterone system inhibitors are commonly prescribed for CKD patients, this association could be tested in our KI mice. This might explain the observed lack of improvement in clinical features such as proteinuria, calciuria, fibrosis or renal function.

## 5 CONCLUSION

In conclusion, 8 months of empagliflozin treatment in KI mice did not improve clinical features such as proteinuria, calciuria, fibrosis, inflammation or renal function. However, SGLT2 inhibition significantly reduced expression of LCN2, supporting its role in the development of Dent's disease.

## AUTHOR CONTRIBUTIONS

Elise de Combiens, Stéphane Lourdel, Yohan Bignon and Marc Fila conceived and designed the project. Elise de Combiens and Stéphane Lourdel conceived and designed the experiments. Elise de Combiens, Nadia Frachon, Clément Brossard and Perrine Frère performed the experiments, with the majority done by Elise de Combiens. Elise de Combiens and Stéphane Lourdel analysed the data. Elise de Combiens and Stéphane Lourdel wrote the manuscript. All authors approved the final version of the manuscript and agree to be accountable for all aspects of the work in ensuring that questions related to the accuracy or integrity of any part of the work are appropriately investigated and resolved. All persons designated as authors qualify for authorship, and all those who qualify for authorship are listed.

## CONFLICT OF INTEREST

None declared.

## Data Availability

All data supporting the results are in the paper.
